# Pregnancy outcomes and early infancy physical growth of fetal situs inversus during the COVID-19 pandemic

**DOI:** 10.3389/fmed.2025.1581322

**Published:** 2025-10-03

**Authors:** Yongke Zhang, Xingsheng Dong, Haijun Li, Ran Chen, Fenge Cai, Dirong Zhang

**Affiliations:** ^1^Department of Obstetrics and Gynecology, Peking University Shenzhen Hospital, Shenzhen, Guangdong, China; ^2^Prenatal Diagnosis Center, Zhongshan Boai Hospital, Zhongshan, Guangdong, China; ^3^Department of Ultrasonography, Peking University Shenzhen Hospital, Shenzhen, China; ^4^Department of Obstetrics, Zhongshan Boai Hospital, Zhongshan, Guangdong, China

**Keywords:** pregnancy, situs inversus, dextrocardia, infant, physical growth, coronavirus disease 2019 (COVID-19), SARS-CoV-2

## Abstract

**Objective:**

This research aimed to observe the pregnancy outcomes and early infancy physical growth of fetuses with situs inversus detected during the coronavirus disease 2019 (COVID-19) pandemic at two centers in South China.

**Methods:**

Data were collected from pregnant women with fetal situs inversus between March 2023 and May 2024. Systemic structured ultrasound, clinical manifestations, and genetic tests were conducted. Data on pregnancy outcomes and routine physical examinations of neonates and infants were collected. Statistical analysis was performed to compare the incidence rate of fetal situs inversus in 2023 with the median incidence rate in previous years. Z-scores for head circumference, weight, and length of the infants were calculated using PediTools Electronic Growth Chart Calculators.

**Results:**

A total of 27 patients with fetal situs inversus were detected; situs inversus with dextrocardia was the most common category of all cases (23/27, 85.2%). The incidence rate of fetal situs inversus in Center 1 increased more than sixfold in 2023 compared with the median incidence rate of previous years (1.75‰ vs. 0.25‰, *p* = 0.000), while in Center 2, the increase was not so obvious (1.22‰ vs. 0.51‰, *p* = 0.011), both with the peak number of affected fetuses detected mainly in April 2023. In total, 15 newborns with situs inversus totalis (SIT) were delivered (15/27, 55.6%), and the majority were alive and reached their normal growth milestones, except for one infant death. Overall, 11 pregnancies underwent therapeutic abortion, and 1 pregnant woman received twin reduction surgery for one of the cotwins (12/27, 44.4%). Z-score evaluation showed no abnormal early physical growth in weight, head circumference, or length among infants with SIT.

**Conclusion:**

A high incidence of fetal situs inversus during the COVID-19 pandemic was observed in our study. Situs inversus with dextrocardia was associated with relatively good pregnancy outcomes in non-complicated cases. No abnormal early infancy physical growth in weight, head circumference, or length was observed among infants with SIT.

## 1 Introduction

The normal arrangement of internal organs is known as situs solitus, while situs inversus represents a rare congenital condition in which the major visceral organs are reversed or mirrored from their normal positions (1). Situs inversus totalis (SIT), also known as situs inversus with dextrocardia, is the most common condition and involves the complete transposition of all of the visceral organs, including the heart. The incidence rate is approximately 1 in 8,000 to 1 in 25,000 live births (2). People with SIT have no associated medical symptoms or complications (1), but the condition may cause some confusion in diagnostic and therapeutic procedures, such as in cases of appendicitis or when organ transplantation is required. Rarely, situs inversus with levocardia occurs when the heart remains in its normal position while the other organs are mirrored, and it carries a high risk of severe heart defects. Even rarer forms, such as situs ambiguous or heterotaxy, involve subsets of organs with abnormal positioning or morphology and are more likely to cause medical problems than SIT. Fetal situs inversus has been described since Aristotle’s time, but its precise cause remains undetermined.

In the past 2 years, researchers from different centers in China (3–6) reported an increase in cases of fetal situs inversus in April 2023, after the “Zero-COVID-19” policy was lifted. Our centers also observed this extremely rare phenomenon. This research aimed to observe the pregnancy outcomes and early infancy physical growth of fetal situs inversus in pregnant women during the COVID-19 pandemic, from March 2023 to May 2024, and to discuss the relationship between the abnormality and SARS-CoV-2 infection.

## 2 Materials and methods

### 2.1 Participants

Pregnant women who were diagnosed with fetal situs inversus during pregnancy and either received routine prenatal care at, or were referred for prenatal diagnosis to, Zhongshan Boai Hospital (Center 1) or Peking University Shenzhen Hospital (Center 2) between March 2023 and May 2024 were included in the study. Those who refused to enroll or could not complete the follow-up visits were excluded from the study. Clinical information such as age, last menstrual period, gravidity and parity history, family history, and teratogenic exposure was collected. Data on fetal situs inversus from 2015 to 2022 at the two centers were retrieved from electronic medical records. The incidence rate of fetal situs inversus in previous years was calculated. The study was approved by the Ethics Committee of Peking University Shenzhen Hospital and Zhongshan Boai Hospital. Written informed consent was obtained from all participating pregnant women for the publication of this study.

### 2.2 Imaging features

A systematic, structured ultrasound scan, including a double check, was conducted by physicians with specialized training appropriate for diagnostic ultrasound in pregnancy. The scan was conducted between 20 and 24 gestational weeks, or at the time the woman presented to the hospital following referral from another center. The ultrasound instruments used were Voluson E10 systems (GE Healthcare, Zipf, Austria), with probe frequencies of 4~8 MHz. To assess cardiac situs, fetal laterality must first be determined, followed by evaluation of the positions of the stomach and heart. Situs abnormalities should be suspected when the fetal heart and/or stomach are not located on the left side. An abnormal cardiac axis increases the risk of cardiac malformations, especially involving the outflow tracts. A systematic, structured ultrasound examination is then performed. Ultrasound features of the fetus were described following the current clinical guidelines (7, 8).

### 2.3 COVID-19 diagnosis and clinical manifestations at early gestational age

The clinical manifestations of upper respiratory infection symptoms at early gestational age and their treatments were collected in the medical record system or by asking the pregnant women on the telephone. SARS-CoV-2 infection and reinfection diagnostic criteria followed the Coronavirus Disease-2019 (COVID-19) 2021 Case Definition of the US CDC^1^ and previous reports (9).

### 2.4 Genetic tests

Since chromosomal abnormalities and gene mutations are common causes of prenatal fetal malformations, amniocentesis was performed on informed pregnant women under the guidance of B-mode ultrasound, and 30 mL of amniotic fluid was extracted. G-banding fetal chromosome karyotype analysis was performed. Chromosomal microarray analysis (CMA) was performed using Affymetrix CytoScan HD arrays (Affymetrix Inc., Santa Clara, CA, United States) according to the manufacturer’s instructions. Data were analyzed using Chromosome Analysis Suite software version 1.2 (Affymetrix Inc., Santa Clara, CA, United States). Trio whole-exome sequencing (trio-WES) was performed using Illumina NextSeq 6,000 (Illumina Inc., San Diego, CA, United States); all reads were aligned with the human reference genome (hg19). Bioinformatics analysis was performed, and pathogenicity was assessed as per the updated American College of Medical Genetics and Genomics (ACMG) guidelines. Candidate variants were confirmed by Sanger sequencing.

### 2.5 Follow-up of the pregnancy outcomes

Pregnancy outcomes of all cases were collected from electronic medical records. Management during pregnancy, delivery mode, gestational age at delivery, and information such as gender, weight, and general condition of the newborns after birth were documented.

### 2.6 Follow-up of early infant physical growth and Z-score evaluation

Early infant physical growth metrics such as head circumference, weight, and length were collected at birth, 1 month, 3 months, and 6 months of age from the participants’ medical files. Z-scores of these growth indicators were calculated using PediTools Electronic Growth Chart Calculators, a widely utilized lambda-mu-sigma-based anthropometric measurement calculation tool, following the operating instructions (10). Z-scores between [−2~2] for head circumference, weight, and length were all indicative of normal growth.

### 2.7 Statistical analysis

Statistical analysis was conducted using SPSS 25.0 software (IBM, Armonk, NY, United States). The comparison of the incidence rate of fetal situs inversus between the year 2023 and the median incidence rate in the previous years (from 2015 to 2022) was performed using the chi-squared test, and a *p*-value of less than 0.05 was considered statistically significant.

## 3 Results

### 3.1 Clinical characteristics of the participants

A total of 27 cases of fetal situs inversus were detected at the two centers (Table 1). The ages of the pregnant women ranged from 25 to 39 years. Three women underwent assisted reproductive technology, while the others had natural conception. No obvious family history or teratogenic exposure was reported. Overall, two women with dichorionic diamniotic twins were included in this study; both of whom had one affected fetus. A total of 23 pregnant women (23/27, 85.2%) experienced upper respiratory infection symptoms during early pregnancy. Fever, especially above 38 °C, was the most common clinical characteristic, along with cough, muscle aches, sore throat, headache, fatigue, anorexia, loss of taste, and stuffy nose. A self-testing SARS-CoV-2 Ag rapid test was performed at the time the women were affected. According to clinical criteria and laboratory criteria, 21 pregnant women (77.8%) were confirmed to have SARS-CoV-2 infection, while two others (7.4%) were diagnosed as probable cases.

**Table 1:** Distributions of the ultrasound characteristics, clinical manifestations at early gestational age, pregnancy outcomes, and neonatal information of all the 27 cases of fetal situs inversus.

Case	Age	G & P	Ultrasound characteristics	Clinical manifestations at early gestational age	Pregnancy outcomes	Infant status at follow-up (May 2024)
1	30	G1P1	SIT	Fever, up to 38.5 °C, and muscle aches lasted for 2~3 days	Cesarean section at 39 wk due to breech presentation; male infant, 3,100 g, was admitted to the NICU for 3 days after birth	8.5 months old, 70.5 cm in length, and 8.7 kg in weight
2	33	G3P2	SIT, intestinal malrotation, and intestinal obstruction	Fever, up to 39 °C, lasted for 3~4 days, cough, fatigue, and anorexia	Therapeutic abortion at 24 wk	/
3	35	G3P2	SIT, ventricular septal defect, and coarctation of the aorta	Low-grade fever, 37 °C	Therapeutic abortion at 23 wk + 3 d	/
4	33	G3P1	SIT	Fever, up to 39 °C, lasted for 1~2 days, cough, muscle aches, sore throat, and headache	Vaginal delivery at 38 wk + 2 d, male, 3,200 g, scalp hematoma at birth, had an intermittent cough	6 months old, 69 cm in length, and 9 kg in weight
5	38	G5P2	Twins, F2 situs inversus with levocardia, atrial inversion, double-outlet right ventricle, severe pulmonary atresia and stenosis, pulmonary dysplasia, ventricular septal defect	Fever, up to 38.5 °C, lasted for 2 days, fatigue, and stuffy nose	Fetal reduction at 23 weeks, and non-affected fetus delivered vaginally at 34 g wk + 2 d due to recurrent preterm labor, female infant, 2,150 g	The non-affected fetus with 9 months old, 65.5 cm in length, 8 kg in weight, intermittent hypersensitivity, and lactose intolerance
6	31	G1P0	Situs inversus with levocardia, complete transposition of the great arteries, and severe ventricular septal defect (functional single ventricle)	Fever, up to 38 °C, lasted for 2 days, cough, sore throat, and stuffy nose	Therapeutic abortion at 18 wk + 3 d	/
7	29	G2P1	SIT	Fever, up to 38.7 °C, cough and night sweats	Vaginal delivery at 38 wk + 4 d of pregnancy, male infant, 3,660 g, was treated with blue light irradiation for jaundice	5 months old, 67 cm in length, and 7.6 kg in weight
8	27	G1P1	SIT	Fever, 37~38 °C, and stuffy nose for 4~5 days, no medicine was taken	Vaginal delivery at 38 wk + 4 d of pregnancy, male infant, 3,500 g, suffered from neonatal jaundice	9 months old, 78 cm in length, and 11 kg in weight
9	32	G3P3	SIT, right ventricular double outlet, and ventricular septal defect	Fever of 38 °C, lasting 2 days, fatigue, cough for 2 weeks, oral Chinese medicine	Vaginal delivery at 39 wk + 1 d, female infant, 3,600 g, had recurrent lung infections after birth, cough and sputum, poor sleep, and prolonged crying	Died of severe pneumonia with multiple organ failure in 5 months old
10	32	G2P1	Situs inversus with levocardia, persistent truncus arteriosus type III, complete ectopic pulmonary venous drainage, and ventricular septal defect	Fever, 37~38 °C, cough for 4~5 days, and dizziness	Therapeutic abortion at 24 wk + 5 d	/
11	39	G3P3	SIT	/	Cesarean section at 38 wk, female infant, 2,580 g	9 months old, 67.5 cm in length, and 8.5 kg in weight
12	39	G3P1	SIT, double-outlet right ventricle, Interrupted aortic arch, and ventricular septal defect	No fever but cough, fatigue, muscle aches and sore throat, no medicine was taken	Therapeutic abortion at 26 wk	/
13	33	G4P2	SIT	Fever up to 38 °C	Vaginal delivery at 38 wk + 2 d, male infant, 2,850 g	9 months, length, and weight not measured recently
14	28	G3P0	Situs inversus with levocardia, left ventricular functional single ventricle, complete atrioventricular septal defect, and pulmonary atresia	Fever up to 38.5 °C, fatigue, sore throat, diarrhea, and ageusia	Therapeutic abortion at 23 wk	/
15	27	G2P1	SIT	Fever up to 38 °C, cough, fatigue, and muscle aches, no medicine was taken	Vaginal delivery at 40 wk, male infant, 2,740 g	8 months old, 69 cm in length, and 9.8 kg in weight
16	26	G2P1	SIT and corrected transposition of the great arteries	Fever up to 39 °C, 3 days, fatigue, sore throat, and muscle aches, oral Chinese medicine was taken	Therapeutic abortion 24 wk	/
17	36	G3P1	SIT, single ventricular atrioventricular septal defect, and parallel ectopic of the great arteries	Fever up to 38.7 °C, 3 days, fatigue, and sore throat	Therapeutic abortion at 21 wk	/
18	36	G2P2	SIT	Fever up to 38.2 °C	Vaginal delivery at 38 wk, male infant, 3,120 g	8 months, 72 cm in length, and 8.7 kg in weight
19	26	G3P2	SIT	Fever up to 38.9 °C, 2 days, acetaminophen tablets were taken	Vaginal delivery at 40 wk, male infant, 2,900 g	8 months old, 71 cm in length, 8 kg in weight, hospitalized due to flu
20	26	G2P2	SIT	Cough, stuffy nose, sore throat, loss of taste, and fatigue	Vaginal delivery at 40 wk, female infant, 3,000 g	5 months old, 65.1 cm in length, and 7 kg in weight
21	46	G4P3	SIT	Fever, up to 38 °C, 2~3 days, stuffy nose, and bupleurum granules were taken	Vaginal delivery at 37 wk + 5 d, male infant, 2,750 g	6 months old, 65 cm in length, 6.6 kg in weight, and night cry occasionally
22	31	G2P2	SIT	Cough, sore throat, and flu-like symptoms lasted for 1 week	Therapeutic abortion at 13 wk	/
23	34	G2P2	SIT and endocardial cushion defect	Fever up to 38.8 °C, headache, and sore throat; oral Chinese medicine was taken	Therapeutic abortion at 22 wk	/
24	33	G2P2	SIT	/	Vaginal delivery at 37 wk + 3 d, male infant, 3,150 g	3 months old, 60 cm in length, and 5.4 kg in weight
25	29	G4P1	Twins, one was SIT	/	Twin pregnancy, cesarean section at 37 wk + 3 d, both females, normal infant 2,650 g, and affected infant 2,840 g	3 months, 58 cm and 59.5 cm in length, respectively, both 5.4 kg in weight
26	25	G2P1	SIT, mitral atresia, ventricular septal defect, and right ventricular double outlet	/	Therapeutic abortion at 18 wk	/
27	35	G1P0	SIT	Fever approximately 37~38 °C	Delivered at 35 wk + 2 d by cesarean section due to hydramnios, female, 1,950 g	5 days after birth, coughing and choking when feeding, a congenital esophageal atresia was confirmed.

G, gravidity; P, parity; SIT, situs inversus totalis; wk, gestational weeks; d, day (s).

### 3.2 Description of the ultrasound characteristics and incidence of fetal situs inversus

Situs inversus with dextrocardia was the most common category among all cases (23/27, 85.2%), including 15 uncomplicated SIT cases (15/27, 55.6%) and 8 cases complicated by heart defects (8/27, 29.6%). Situs inversus with levocardia was found in the remaining 4 cases (4/27, 14.8%), all of which were combined with severe heart defects. Right ventricular double outlet was the most common ultrasound abnormality (4/12, 33.3%) in all the cases. The incidence rate of fetal situs inversus was compared with data from the previous years in the two centers. The results revealed that the incidence rate of fetal situs inversus in Center 1 increased more than sixfold in 2023 compared to the median incidence rate of the previous years (1.75‰ vs. 0.25‰, *p* = 0.000), while in Center 2, the increase was not so obvious (1.22‰ vs. 0.51‰, *p* = 0.011) (Table 2), both with the peak number of affected fetuses mainly detected in April 2023, which coincidentally overlaps with the time of early pregnancy infection of SARS-CoV-2.

**Table 2:** Comparison of the incidence rate of fatal situs inversus between the year 2023 and the years from 2015 to 2022.

Group	Incidence of fetal situs inversus		X^2^	*p*
	Median in year 2015~2022	2023		
Center 1	0.251 (20^a^/79,590^b^)	1.745 (15/8,597)	43.624	0.000
Center 2	0.513 (47/91,556)	1.216 (10/8,223)	6.527	0.011

^a^Case number of fatal situs inversus; ^b^Total pregnancy.

### 3.3 Follow-up of pregnancy outcomes

Overall, 15 newborns with situs inversus were delivered (15/27, 55.6%), including 11 male and 4 female newborns. Of these, 11 newborns were delivered vaginally and 4 by cesarean section. Only one pregnancy was delivered by cesarean section at 35 gestational weeks due to hydramnios, and the newborn was diagnosed with esophageal atresia after birth. A total of 14 newborns were stable at birth and thereafter. One infant who had complications such as right ventricular double outlet and ventricular septal defect died of severe pneumonia with multiple organ failure at 5 months of age. In total, 11 pregnancies underwent therapeutic abortion, and 1 pregnant woman received twin reduction surgery for one of the cotwins due to a severe fetal heart defect (12/27, 44.4%). A total of 8 women underwent amniocentesis in the second trimester, but fetal chromosome karyotyping, CMA, and trio-WES did not reveal positive results.

### 3.4 Early infant physical growth at follow-up

All newborns were rechecked using ultrasound, confirming the prenatal diagnosis of situs inversus. By and large, they reached normal growth milestones at 1 month, 3 months, and 6 months of age during follow-up. No long-term respiratory symptoms were reported during their early lives. Except for one infant with esophageal atresia and another who had intermittent hypersensitivity and lactose intolerance after birth, no other severe or sustained symptoms or diseases were found in the infants during follow-up. Z-score evaluation using anthropometric measurement calculation tools showed that there was no significant abnormal early infancy physical growth in weight, head circumference, and length of the infants with non-complicated situs inversus totalis (Supplementary Table 1). Only one infant (Case 8) had a length Z-score of 2.98 at 6 months of age; all other anthropometric measurements fell into the normal range.

## 4 Discussion

Studies indicated that motile cilia dysfunction expressed early in development is involved in left–right body axis determination (11, 12). Cilia are hair-like organelles that protrude from the cell surface into the extracellular space and are widely expressed in human tissues, including the respiratory epithelium (13). Primary ciliary dyskinesia (PCD), an autosomal recessive genetic condition, is a disease caused by dysfunction of the cilia that occurs during early embryonic development (14). Normally functioning cilia determine the position of the internal organs during early development, so embryos with PCD have a high risk of developing situs inversus. Cilia are also responsible for clearing mucus from the lungs, and their dysfunction causes increased susceptibility to lung infections, which are often observed in infants shortly after birth or at an early age. Mutations in approximately 50 cilia-related genes are associated with PCD, including *DNAI1*, *DNAH5,* and *DNAH11* (15). However, in our cases, among those who underwent amniocentesis and trios-WES, no pathogenic gene mutations were detected. Therefore, other factors besides gene mutations must have contributed to the progression of early embryonic developmental abnormalities.

A marked increase in cases was observed several months after the lifting of the “Zero-COVID-19” policy in China (3–6), which coincided with an increase in SARS-CoV-2 infections. SARS-CoV-2 has been observed to cause defects in the ciliated layer of bronchial epithelial cells in deceased COVID-19 patients and animal models (16). This rare clinical evidence suggests a possible link between infection during pregnancy and the development of situs inversus in the fetus, especially during gestational weeks 4 ~ 6, the critical period for organ positioning.

In 2021, the World Health Organization (WHO) added SARS-CoV-2 to the list of vertically transmissible infectious agents. The vertical transmission of COVID-19 in the third trimester is approximately 3.2% by infant nasopharyngeal swab testing, with SARS-CoV-2 RNA positivity in 7.7% by placental sample analysis (17). Studies indicate that SARS-CoV-2 can infiltrate the placenta and infect the fetus at different pregnancy stages, but the precise mechanisms of intrauterine transmission remain unclear. An angiotensin-converting enzyme 2 (ACE2)-associated transmission model may explain the progress of placental translation. ACE2, a placental cell receptor, together with the consistently present neuropilin-1 (NRP-1), can change expression during pregnancy and mediate SARS-CoV-2 infiltration of the placenta, breaching its protective barrier, and infecting the fetus (18). Xiuli Shao et al. confirmed that the expression of ACE2 in ventricular myocytes was upregulated in both fetuses and adults with heart damage (19). If the fetus were infected with the virus, embryopathies and congenital anomalies could result.

However, increased cases of fetal situs inversus after SARS-CoV-2 infection were not seen in the United States (20) and Scandinavian countries (21). The reason for this may be due to the strict lockdown policy by the Chinese government after the outbreak of the pandemic, which protected the majority of Chinese people against infection. The vast majority of people were first infected within a short time window in December 2022. Therefore, China represents a unique model worldwide, making it advantageous for observing complications of the disease. In our study, we observed a significant increase in cases of fetal situs inversus in one prenatal diagnostic center, but data from the other center were not fully consistent with it. This discrepancy was mostly because Center 2 was a regional fetal abnormality referral center, which accepted patients from surrounding cities and thus had a relatively high incidence of fetal situs inversus in the previous years.

A series of structural abnormalities complicated by SIT were detected in our study, such as double-outlet right ventricle, endocardial cushion defect, pulmonary stenosis, pulmonary atresia, single ventricle, transposition of the great arteries, and ventricular septal defect. Early diagnosis and treatment are needed due to the severe complications of specific abnormalities. Situs inversus with levocardia usually carries a high risk of severe heart defects; thus, therapeutic abortion or urgent surgery after birth may be required. In cases of SIT, our study supports that individuals with this abnormality may have no medical symptoms or complications.

Infants of SIT without severe ultrasound abnormalities in our study showed normal physical growth and development at follow-up. The infant of Case 5 had intermittent hypersensitivity and lactose intolerance after delivery, but the syndrome is common in neonates and resolved spontaneously at follow-up visits. Case 8 had a Z-score of 2.98 at 6 months of age, which was outside the normal range; further analysis revealed no other causes, and the deviation was attributed to the parents’ height.

This study only contained a small number of participants, which may make statistical analysis difficult. Clinical information collection was mostly via telephone visits; thus, recall bias was inevitable. Meanwhile, the physiological immunosuppression of pregnant women, the limited transfer of anti-SARS-CoV-2 antibodies from the mother to the fetus, side effects of drugs or vaccines used during the pandemic, and other unknown causes were also indicated as limitations of the study.

## 5 Conclusion

A high incidence of fetal situs inversus during the COVID-19 pandemic was observed in our study. Situs inversus with dextrocardia was the most common condition of fetal situs inversus and had a relatively good pregnancy outcome in non-complicated cases. Abnormal early infancy physical growth in weight, head circumference, and length of the infants with SIT was not observed in the study.

## Data Availability

The raw data supporting the conclusions of this article will be made available by the authors, without undue reservation.

## Footnotes

^1^ https://ndc.services.cdc.gov/case-definitions/coronavirus-disease-2019-2021/
